# Neurobiological Mechanisms of Pelvic Pain

**DOI:** 10.1155/2014/903848

**Published:** 2014-07-08

**Authors:** Massimo Origoni, Umberto Leone Roberti Maggiore, Stefano Salvatore, Massimo Candiani

**Affiliations:** Obstetrics and Gynecology Unit, Vita-Salute San Raffaele University and IRCCS San Raffaele Hospital, Via Olgettina 58-60, 20132 Milan, Italy

## Abstract

Pelvic pain is a common condition which significantly deteriorates health-related quality of life. The most commonly identified causes of pain in the pelvic region are gynaecologic, urologic, gastrointestinal, neurological, and musculoskeletal. However, in up to 33% of patients the source of this symptom is not identified, frustrating both patients and health-care professionals. Pelvic pain may involve both the somatic and visceral systems, making the differential diagnosing challenging. This paper aimed to review the mechanisms involved in pelvic pain perception by analyzing the neural plasticity and molecules which are involved in these complex circuits.

## 1. Introduction

Pelvic pain is a main issue for patients and health care systems. It not only significantly deteriorates the quality of life of patient himself/herself, but also affects the relationships with the partner, relatives, and colleagues thus representing a consistent social burden. Furthermore, pelvic pain is frustrating not only for patients but also for physicians, who try to identify a pathologic reason in order to justify the clinical condition. In addition, even when anything “abnormal” is diagnosed, it does not necessarily mean that it represents the cause of pain symptoms referred by the patient. However, in common clinical practice, the physician may be not able to diagnose the source of the complaints in up to 33% of cases [1]. In the absence of a conclusive diagnosis, patients are commonly and erroneously labelled with a psychogenic cause of their pain, thus further increasing patient's disappointment [2].

Pelvic pain in women is a very common and debilitating complaint. In particular, chronic pelvic pain (CPP) is estimated to have a prevalence between 4 and 15% [3, 4], accounting for 10% of all ambulatory referrals to a gynecologist, representing the indication for 12% of all hysterectomies and for over 40% of all gynecological diagnostic laparoscopies [3]. Patients frequently suffer from pelvic pain throughout their reproductive ages, for very long periods of time; more rarely a premenarche or postmenopause onset is referred [3, 4].

Several causes may be at the basis of pelvic pain; however, not always an organic disorder is present as a pain cause (Table 1). In this case pain perception is usually due to a “functional pathology,” often with a neuropathic basis. As a consequence, only few physicians, specialized and trained in pelvic pain disorders, can easily recognize what the etiology of pelvic pain is, and the treatment is often exclusively symptomatic, frequently with less efficacy.

The mechanisms underlying the onset and the persistence of pelvic pain, are linked to the existence of complex circuits, which involve peripheral neural pathways, the spinal cord, and brain areas. There are numerous interconnections among the nervous system and the anatomical structures of the pelvis and through the different pelvic organs themselves. The study of this extended network of communication and mechanisms of pain perception has fascinated and continues to fascinate many scientists and clinicians, despite remaining largely unexplained. The current paper aimed to review the mechanisms involved in pelvic pain perception by analyzing the neural plasticity and molecules which are involved in these complex circuits.

## 2. Nerve Supply of the Pelvis

**Figure 1:**
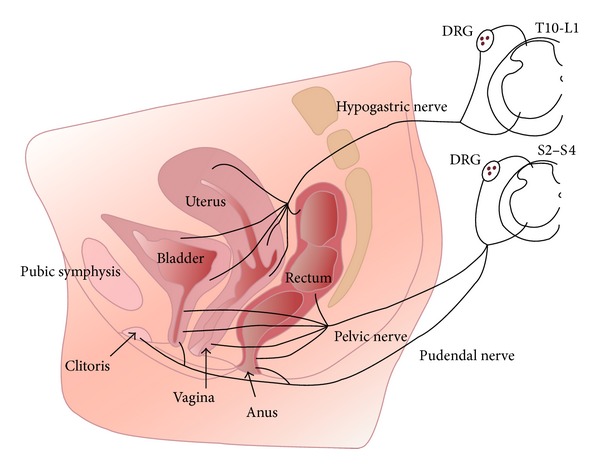
Innervation of pelvic organs. Sensory axons innervating the vagina reach the spinal cord via pelvic nerves and terminate in sacral spinal cord segments (S2-S4). Axons innervating the uterus travel in the hypogastric nerves and terminate in the thoracolumbar spinal cord segments (T10-L2). The region surrounding the cervix represents a transitional zone and is innervated by fibers that travel in both nerves. Sensory axons from the clitoris and vulva follow the pudendal nerves to sacral spinal cord. Note that sensory information from all pelvic organs may converge on to the same spinal cord neural circuits. DRG (dorsal root ganglia). Reproduced from Jobling et al., 2014, with permission [61].

The anatomical structures that may give rise to pain in the pelvic region belong to the urinary, reproductive, and gastrointestinal systems and to their associated blood and lymphatic vessels. These structures, innervated by the somatic (T12-S5) and visceral (T10-S5) nervous system, create a complex anatomical and neurobiological network (Figure 1).

Dual projection from the thoracolumbar and sacral segments of the spinal cord carries out this innervation, converging primarily into discrete peripheral neuronal plexuses and then distributing nerve fibers throughout the pelvis. The visceral afferents travelling the sympathetic trunk have cell bodies in the thoracolumbar dorsal root ganglia (DRG), and those that travel with the parasympathetic fibers have cell bodies in the sacral DRG. The thoracolumbar and sacral DRG cells are the first of numerous relays of sensory neurons that transmit painful sensations from the pelvis to the brain. Studies in animal models suggest that sensations from the pelvis are conveyed within the sacral parasympathetic system, with a lesser contribution from the sympathetic thoracolumbar system [5].

The hypogastric plexus is the main autonomic neuronal center of the pelvis, while the somatic innervation is guaranteed by fibers travels in the pudendal nerve. This is the main nerve of the pelvis, involved in a great amount of pelvic pain conditions. It contains also sympathetic and parasympathetic efferents and visceral afferents. The pudendal nerve originates from the sacral plexus (S2-S4), then exits the pelvis through the greater sciatic foramen into the perineal area, through the pudendal (Alcock's) canal, and finally spreads into three main terminal branches: the inferior rectal nerve, the superficial perineal nerve, and the dorsal nerve of the clitoris, which innervates the pelvic structures and the external genitalia. In addition to sensory branches, the pudendal nerve provides motor innervation to anal and urethral sphincters, as well as to the bulbospongiosus and ischiocavernosus muscles (involved in the bulbocavernosus response and orgasm).

Injury to the pudendal nerve or its branches can cause chronic pain in the innervated regions, for example, as a result of compression of the nerve in the pudendal canal (from prolonged labor or straining with stools) or from cutting injury during a mediolateral episiotomy.

## 3. Pain and Nociception

### 3.1. Pain Definition

Pain and nociception do not have the same meaning. As a matter of fact, while nociception represents the process of transmitting to centers involved in perception information about a stimulus that has the potential to cause tissue damage; the definition of pain not only includes the pathways involved in stimulus transmission, but also involves an emotional response. The International Association for the Study of Pain (IASP) defines pain as “an unpleasant sensory and emotional experience associated with actual or potential tissue damage or described in terms of such damage” [6]. A revised definition identifies pain as “a somatic perception containing a bodily sensation with qualities like those reported during tissue-damaging stimulation; an experienced threat associated with this sensation; and a feeling of unpleasantness or other negative emotions based on this experienced threat” [7]. However, despite the definition, it is clear that pain is a subjective sensation and that it is the “unpleasantness” of the pain experience that makes it an emotional experience and not simply a sensory one.

### 3.2. Types of Pain

Pelvic pain, like other types of pain, can be classified based on the duration of pain (acute or chronic) or on the basis of the underlying biological mechanism.

Acute pain represents a vital and protective mechanism that allows the individual to react against potential dangerous stimuli [8]; it may be described as an adaptive response. On the other hand, chronic pain is a maladaptive and pathologic functioning of the nervous system. Clinically it is described as “noncyclic pain of 6 or more months duration that localizes to the anatomic pelvis, abdominal wall at or below the umbilicus, lumbosacral back or the buttocks and is of sufficient severity to cause functional disability or lead to medical care” [9]. Chronic pelvic pain is commonly associated with negative cognitive, behavioural, sexual, and emotional consequences.

The biological mechanisms underlying pelvic pain conditions may be nociceptive (1), inflammatory (2), neuropathic (3), psychogenic (4), mixed (5), or idiopathic (6). The T10-L1 afferent visceral pain fibers that innervate the uterus, adnexa, and cervix also supply the lower ileum, sigmoid colon, and rectum; thus, pelvic pain sensations can originate in any of those closely related structures.

(1) Nociceptive pain is often thought of as “normal pain.” It is provoked by the activation of nociceptors by noxious stimuli or by stimuli that would become noxious if prolonged. It may be visceral or somatic. In pelvic pain, nociceptive pain is usually visceral and results from pelvic organs distension, ischemia, or spasm, secondary to thermal, chemical, and mechanical stimuli. Deep visceral pain is poorly localized and has some overlap with somatic sensory tracts in the spinal cord, causing “referred pain.”

(2) Inflammatory pain is due to the response to tissue injury and the resulting inflammatory process that may also activate “silent nociceptors,” which do not normally respond to intense mechanical or thermal stimuli. This mechanism is probably involved in women with pelvic endometriosis, because many visceral nociceptors, especially silent nociceptors, may only respond to mechanical stimuli when inflammatory tissue disease, such as endometriosis, is present [10].

(3) Neuropathic pain, usually described as a burning and tingling pain, arises from abnormal neural activity secondary to disease, injury, or dysfunction of the nervous system. It commonly persists without ongoing disease. This type of pain may be subdivided into sympathetically mediated pain (if a peripheral nerve lesion exists, associated with autonomic changes), peripheral neuropathy (if a damage to a peripheral nerve exists, without autonomic modifications), and central pain (due to the alterations in the central nervous system which occur in chronic pain disorders).

Furthermore, the etiology of pelvic pain may be also (4) psychogenic, as a result of the physical manifestation of unresolved emotional or psychological conflict.

Finally, mixed cause of pain occurs when the previous mechanisms overlap, while pain is defined as idiopathic when it is not possible to identify an etiological factor.

When chronic pelvic pain occurs, the main mechanisms involved in pain perception are the neuropathic and the inflammatory one [10].

### 3.3. Pathogenesis of Pelvic Pain

When a noxious or potentially noxious stimulus acts inducing tissue damage, specialized nociceptors localized at the nerve terminals of the primary afferent fibers activate. These stimuli are transduced into electrical impulses which are transmitted via A-delta (fast myelinated) and C-fibers (slow unmyelinated) DRG neurons to synapses in the dorsal horn of the spinal cord, mainly, via the hypogastric plexus and pudendal nerves. These nerves convey sensory information from major pelvic organs: colon, rectum, urinary bladder, and uterus [11]. The majority of afferent DRG neurons project contralaterally within the spinal cord and ascend within the anterolateral quadrant, forming the spinothalamic tract which synapses to the thalamus. Neurons from the thalamus project to multiple brain areas in the primary and secondary somatosensory cortex, cingulated cortex, prefrontal cortex, insular cortex, amygdala, and cerebellum. These areas are responsible for pain perception, the decoding of afferent input that gives rise to individual's sensory experiences. Brain circuits and spinal cord descending pathways can modulate pain afferent pathways altering the sensory input (augmentation/suppression) at its entry “gate,” a process that helped explain how pain could vary in different circumstances [12].

In this regard, it has been demonstrated that descending pain facilitation from the rostral ventromedial medulla plays a crucial role in hyperalgesia, which occurs when stimuli that are normally noxious may be magnified. Descending influences on spinal nociceptive processing primarily involve the periaqueductal grey and the rostral ventromedial medulla, which seems to be the final common output for descending influences from rostral brain sites. However, the rostral ventromedial medulla can also have facilitatory effects on spinal nociceptive transmission. This bidirectional central control of nociception not only may alleviate pain in situations where antinociception is necessary for survival but also could facilitate nociceptive processing and thereby contribute to the maintenance of hyperalgesic states following peripheral tissue damage [13].

## 4. Mechanisms of Persistent/Chronic Pain

### 4.1. Cross-System, Viscerovisceral Interactions

**Figure 2:**
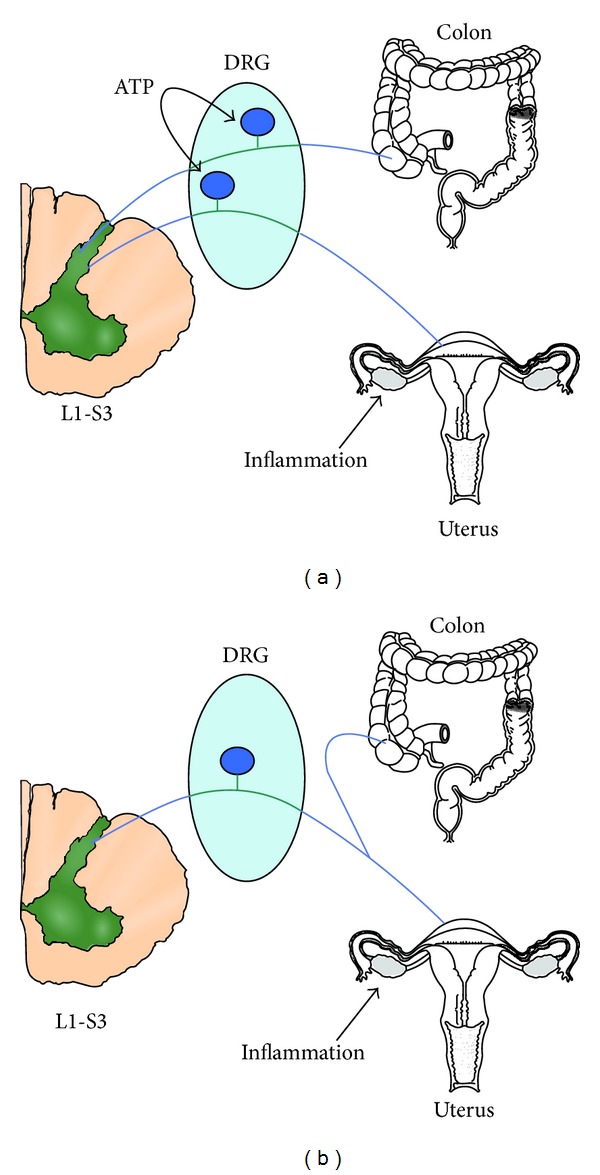
Models of alternative possibilities for viscerovisceral cross-sensitization in the DRG neuron. (a) ATP released by a neuron innervating the inflamed uterus acts on a neighboring neuron sensitizing its responses to colonic distention. (b) The same neuron innervates the uterus and colon. Uterus inflammation directly sensitizes the neuron to colonic distention. Reproduced from Chaban, 2012, with permission [62].

The term “visceral cross-sensitization” refers to the transmission of a noxious stimulus from a diseased pelvic organ to an adjacent normal structure, resulting in functional changes in the latter, and is one of the main factors contributing to development of chronic pelvic pain. These viscerovisceral interactions and reflexes between gastrointestinal, urinary, and reproductive systems are mediated by convergence of sensory information via both the peripheral and central mechanisms of stimulus processing (Figure 2) [14]. Major peripheral mechanisms involve the neurons localized within DRG, whereas central mechanisms include structures in the spinal cord and brain [15].

Despite the fact that cross-talk mechanisms are not well known, three interconnected neural pathways have been proposed.

(1) The first mechanism has been described by Malykhina et al. [16]. It occurs when a painful stimulus from a damaged tissue propagates toward the respective DRG. If the same neuron has axonal connection with another pelvic structure, the impulses may be propagated antidromically inducing releasing of neuropeptides at the side of that organ by provoking hyperalgesia and contributing to “neurogenic inflammation.” This pathway is due to the presence of neuronal cell bodies giving rise to multiple or branching axons. It was demonstrated that the percentage of DRG neurons with multiple or dichotomizing axons lies between 3 and 10% [17–20] and is observed to be present in a higher number in the distal colon and urinary bladder with respect to other organs and tissues, especially in the T13-L2 and L6-S2 DRG neurons [16, 21, 22].

(2) The second pathway refers to the convergence of sensory inputs from normal and diseased structures to the same spinal interneuron located in the dorsal horn of the spinal cord. Different studies in animal models have demonstrated the existence of the “viscera-visceral convergence of sensory neurons” [23, 24]. The percentage of neurons in the spinal cord which receive convergent input from two or more pelvic organs appears to be higher than the proportion of DRG cells innervating multiple viscera. For example, 26% out of all recorded neurons in the dorsal horn of lumbosacral segments of the spinal cord receive afferent inputs from both the colon and urinary bladder.

(3) The third mechanism involves the higher centres of the brain, which can modulate by descending pathway pain perception. In this regard, several studies have documented a role for the neurons of the spinal cord [25] and medulla (nucleus gracilis [26] and solitary nucleus [27], reticular substance [28], and periaqueductal gray) in the modulation of pelvic pain perception and development of hyperalgesia. At the pontine level an important role is played by Barrington's nucleus [29], while in the brain it is played by the lateral thalamus [30, 31] and amygdale [32, 33].

A contribution to the effects caused by SNC in the regulation of pain perception derived also from the psychological and behavioural influences. Stress and anxiety, as a consequence to pelvic pain persistence, play a relevant role in the modulation of central descending pathways [34].

Different studies demonstrated the existence of “viscera-visceral interaction” and their physiopathological consequence. For instance, inflammation of the urinary bladder significantly decreases uterine contraction rate in rats [35], whereas colon and uterine inflammation produced signs of inflammation in the bladder, an effect that involved the hypogastric nerve [36]. Another confirmation of visceral cross-talk was described by Giamberardino et al. who demonstrated that endometriosis not only produced vaginal hyperalgesia but also exacerbated the pain behaviours induced by a ureteral stone and increased bladder motility of an otherwise healthy bladder [37]. These results showed potent cross-system, viscera-visceral interactions in which pathophysiology in one organ influenced the physiology and response to pathophysiology of another organ. The implications of these results for the clinic are likely profound; in showing that pathological events occurring in one organ had significant effects on other organs, even if the other organ was healthy, the results implied that these interactions are part of the processes that bring about the cooccurrence of pain conditions and contribute to dangerous conditions in which pain fails to occur with pathology.

### 4.2. Neurogenic Inflammation and Peripheral Sensitization

**Figure 3:**
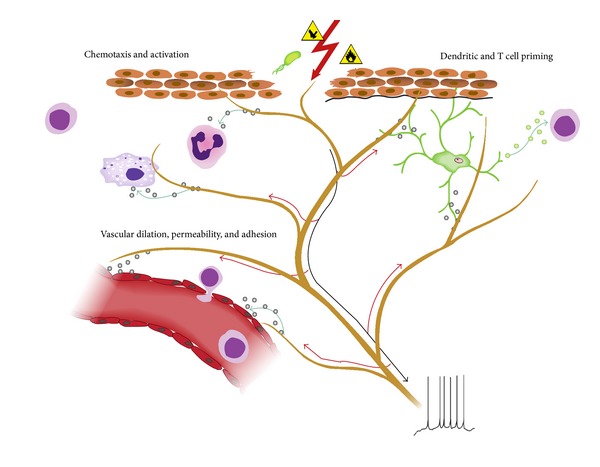
Neurogenic inflammation. Neuronal factors released from nociceptor sensory neurons directly drive leukocyte chemotaxis, vascular hemodynamics, and the immune response. When noxious stimuli activate afferent signals in sensory nerves, antidromic axon reflexes are generated that induce the release of neuropeptides at the peripheral terminals of the neurons. These molecular mediators have several inflammatory actions: (1) chemotaxis and activation of neutrophils, macrophages, and lymphocytes to the site of injury and degranulation of mast cells. (2) Signaling to vascular endothelial cells to increase blood flow, vascular leakage, and edema. This also allows easier recruitment of inflammatory leukocytes. (3) Priming of dendritic cells to drive subsequent T helper cell differentiation into Th2 or Th17 subtypes. Reproduced from Chiu et al., 2012, with permission [63].

Bayliss is the first who demonstrated the existence of antidromic conduction in afferent fibers [38]. In this case the sensory impulses do not travel towards the spinal cord but are directed in the opposite site. When they arrive in the periphery of the area innervated by the primary afferent nociceptors, “neurogenic inflammation” occurs with degranulation of mast cells [39], arteriolar vasodilation, and plasma extravasation (Figure 3) [40]. For example, clinical studies reveal a fourfold increase in the number of mast cells in the urinary bladder and a threefold elevation in the colon in biopsies of patients diagnosed with both interstitial cystitis/pain bladder syndrome (IC/PBS) and inflammatory bowel diseases (IBS) [41].

Calcitonin gene-related peptide (CGRP) and substance P (SP) are the main neuropeptides responsible for the development of neurogenic inflammation in the pelvis [42, 43]. They are highly expressed in primary afferent neurons projecting to the urogenital tract and distal colon [44] and are responsible for edema, vasodilation, and mast cells degranulation. As a matter of fact, it has been demonstrated that experimental colitis results in a 60% decrease of SP protein in L4-S4 dorsal root ganglion neurons projecting to the colon [45], probably due to stimulation of SP release from extrinsic neurons. Furthermore it was described how, after TNBS-induced colonic inflammation, the expression of CGRP in L1 bladder dorsal root ganglion neurons and S1 bladder afferents significantly increased, respectively, by 23% and 11% [46].

It is well known that neurogenic inflammation is associated with the activation of nociceptors sensitive to capsaicin and its analog resiniferatoxin [47], and SP and CGRP very often coexist with the expression of these receptors: the transient receptors potential vanilloid 1 (TRPV1). Urinary bladder inflammation leads to upregulation of TRPV1 in sensory afferents contributing to the development of bladder hyperexcitability and hyperalgesia [48]. Furthermore, experimental colitis significantly enhances the response of bladder afferent neurons to capsaicin by approximately 60% in lumbosacral dorsal root ganglion neurons [49].

Visceral “cross-talk” is also influenced by nerve growth factor (NGF) expression. Animal studies with induced cystitis in rats showed an increased concentration of NGF and its content is often increased in the urine samples of individuals diagnosed with IC/PBS, sensitizing the afferent nerves and inducing bladder hyperactivity [50, 51].

The release of proinflammatory substances at the site of injury such as bradykinin, tachykinins, prostaglandins, serotonin (5-HT), ATP, and protons and the development of “neurogenic inflammation” are responsible for peripheral sensitization. In this context, it was described how CGRP did not provoke pain in healthy subjects but this occurs in those with pelvic pain symptoms which were significantly exacerbated [52].

In fact, the peptides released induce primary afferent fibres activation, sensitization, and recruitment of the “silent nociceptors,” conditioning an increased input to second-order neurons in the dorsal horn and determining hyperalgesia. They can also lower the threshold for activation by normally active stimuli and can activate local immunocytes and/or mast cells with NGF releasing. This induced changes in the distribution of receptors of algogenic mediators and enhanced the expression of sodium channels contributing to peripheral sensitization mechanisms [53, 54].

### 4.3. Central Sensitization

Central sensitization is defined as “an increase in the excitability of the CNS so that normal inputs now evoke exaggerated responses” [55]. Central sensitization occurs when a noxious event in peripheral bodily tissues triggers a long-lasting sensitization of recipient spinal neurons that can continue long after the initial peripheral injury or pathology has healed.

Peripheral sensitization represents the beginning of central sensitization which is maintained by persistent stimulation of the CNS from sensitized sensory afferent fibers. More commonly, the elimination of the algic input causes the resolution of pain perception. However, less frequently, pain persists after the resolution of the cause of pain. Neural mechanisms similar to those underlying memory may cause a process of central sensitization, independent of peripheral sensitization [56–59]. The pain then remains long after the initiating pathophysiology resolves. In these conditions, therapies targeted at the periphery may fail to relieve pain.

The molecular mechanisms involved in the development of central sensitization are incompletely understood. The release of excitatory amino acids and the neuropeptides substance P and CGRP from the central terminals of primary afferent fibres appear to play an important role in the observed central changes. Roles for calcium fluxes through the N-methyl-D-aspartic acid (NMDA) and aminomethylene phosphonic acid (AMPA) receptors channel, nitric oxide, and the expression of protooncogens such as c-fos and c-jun in spinal dorsal horn neurons have been demonstrated [57]. The calcium inflow obtained by NMDA and AMPA receptor activation acts to lower the threshold for second-order neuron firing, with increased signalling being transmitted to the higher centres, and induces posttranslational processing; this usually involves the addition of phosphate groups to amino acids by kinases. Phosphorylation can alter the properties of a protein, not only lowering the threshold at which channels open, but also maintaining the channels open for a longer time. The result is that a stimulus produces a magnified evoked response in these neurons. Pharmacological studies suggest that there is cooperation between substance P- and NMDA-mediated events in the development and maintenance of inflammation-induced central sensitization [53].

The result of these central influences on pain perception causes an expansion of the receptive field and an increasing of signals directed to the CNS, with the amplification of what is perceived from a peripheral stimulus. These phenomena lead to allodynia (which is that stimulus is perceived as painful even if it is not) and hyperalgesia. As a consequence, one can see that many of the symptoms of pelvic pain conditions may be explained by central sensitization.

## 5. Conclusions

Different biological mechanisms may explain the origin of pelvic pain, such as nociceptive, inflammatory, neuropathic, psychogenic, mixed, or idiopathic origin. Noxious stimulus induces the activation of specialized nociceptors and is transmitted via primary afferent fibers to synapses in the dorsal horn of the spinal cord, mainly, via the hypogastric plexus and pudendal nerves. This information is sent within the spinal cord to the thalamus and finally to multiple brain areas which are responsible for pain perception.

Pelvic pain is a troublesome clinical condition and its management is actually challenging for general physicians and specialists. It significantly deteriorates patient's capacity to function in family, sexual, social, and occupational roles. Although a number of causes are known as responsible for pelvic pain, in up to 33% of patients, the source of this symptom is not identified, frustrating both patients and health-care professionals. Commonly, there is not only one cause that may justify the algic symptoms, but also a multifactorial etiology. Taking this into consideration, a multidisciplinary approach should be considered since it is related to significantly better results than those observed after traditional treatment by a single specialist approach [60]. Achieving a strong patient-physician relationship is of primary importance in the management of pelvic pain. In fact, frequently nonsurgical and surgical strategies are not immediately effective in relieving pain and symptoms of depression and both the physician and the patient have to face a long and articulate path to obtain clinical improvements.

## Figures and Tables

**Table 1:** Most common causes of pelvic pain.

*Gynaecologic* (i) Endometriosis/adenomyosis (ii) Pelvic congestion syndrome (iii) Chronic pelvic infections (iv) Uterine myomas	

*Urologic* Painful bladder syndrome	

*Gastrointestinal* (i) Irritable bowel syndrome (ii) Inflammatory bowel diseases	

*Musculoskeletal* (i) Fibromyalgia (ii) Pelvic floor myalgia (piriformis syndrome)	

*Other* (i) Neuralgia of pudendal nerve (ii) Neuralgia of iliohypogastric, ilioinguinal, or genitofemoral nerve (iii) Depression (iv) Visceral hyperalgesia (v) Somatisation disorders (vi) Psychosexual dysfunction (including previous or current sexual abuse) (vii) Porphyria	
